# Usefulness of 3-Dimensional Flexible Endoscopy in Esophageal Endoscopic Submucosal Dissection in an *Ex Vivo* Animal Model

**DOI:** 10.1155/2019/4051956

**Published:** 2019-11-03

**Authors:** Kazutoshi Higuchi, Mitsuru Kaise, Hiroto Noda, Go Ikeda, Teppei Akimoto, Hiroshi Yamawaki, Osamu Goto, Nobue Ueki, Seiji Futagami, Katsuhiko Iwakiri

**Affiliations:** Department of Gastroenterology, Nippon Medical School, Tokyo 113-8603, Japan

## Abstract

**Background and Aims:**

Three-dimensional (3D) rigid endoscopy has been clinically introduced in surgical fields to enable safer and more accurate procedures. To explore the feasibility of 3D flexible endoscopy, we conducted a study comparing 2-dimensional (2D) and 3D visions for the performance of esophageal endoscopic submucosal dissection (ESD).

**Methods:**

Six endoscopists (3 experts and 3 trainees) performed ESD of target lesions in isolated porcine esophagus using a prototype 3D flexible endoscope under 2D or 3D vision. Study endpoints were procedure time, speed of mucosal incision and submucosal dissection, number of technical adverse events (perforation, muscle layer damage, and sample damage), and degree of sense of security, fatigue, and eye strain.

**Results:**

Procedure time and speed of mucosal incision/submucosal dissection were equivalent for 2D and 3D visions in both experts and trainees. The number of technical adverse events using 2D vision (mean [standard deviation], 3.5 [4.09]) tended to be higher than that using 3D vision in trainees (1.33 [2.80]; *P* = .06). In experts, 2D and 3D visions were equivalent. The degree of sense of security using 3D vision (3.67 [0.82]) was significantly higher than that using 2D vision (2.67 [0.52]) in trainees (*P* = .04), but was equivalent in experts. The degree of eye strain using 3D vision (3.00 [0.00]) was significantly higher than that using 2D vision (2.17 [0.41]) in trainees, but was equivalent in experts.

**Conclusions:**

3D vision improves the sense of security during ESD and may reduce technical errors, especially in trainees, indicating the feasibility of a clinical trial of ESD under 3D vision.

## 1. Introduction

To date, therapeutic endoscopy, including endoscopic submucosal dissection (ESD), is performed with a 2-dimensional (2D) flexible endoscope. Endoscopists find it difficult to handle an ESD knife when the target lesion is facing perpendicular to a 2D endoscope, which cannot provide depth information. Misrecognition of the distance between the ESD knife and the target lesion leads to unexpected cutting and dissection and may elicit technical errors, such as perforation of the gastrointestinal tract or damage to the ESD sample. Three-dimensional (3D) visualization offers better depth recognition, and may avoid procedural errors due to misrecognition, and enhances the efficacy and accuracy of therapeutic endoscopy. 3D rigid endoscopes, such as laparoscopes, have already been introduced in surgical fields to enable safer and more accurate procedures [1, 2].

We have already reported that a 3D flexible endoscopy system improves diagnostic accuracy for superficial gastrointestinal neoplasias [3], and have also reported the feasibility of 3D endoscopy in *ex vivo* gastric ESD [4]. In this study, exploring the feasibility of using a 3D flexible endoscope in therapeutic endoscopy, we conducted an *ex vivo* comparison study in isolated porcine esophagus between 2D and 3D visions for the performance of esophageal ESD using a newly developed 3D flexible endoscopy system.

## 2. Methods

### 2.1. 3D Flexible Endoscopy System

**Figure 1:**
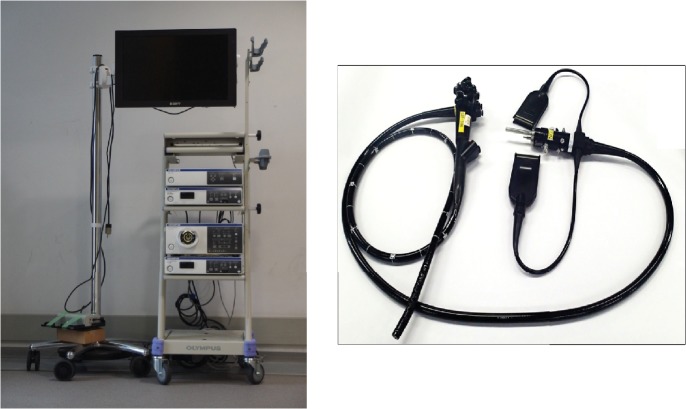
The 3-dimensional endoscopy system.

**Figure 2:**
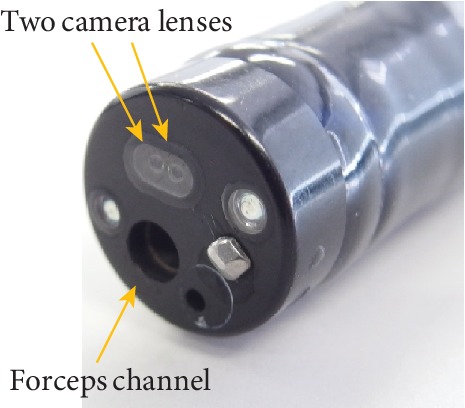
The tip of the 3-dimensional flexible endoscope.

The 3D flexible endoscopy system is composed of a prototype 3D flexible endoscope (GIF-Y0080, Olympus Medical Systems R&D, Tokyo, Japan), two video system centers (EVIS EXERA III Video System Center, CV-190; Olympus Medical Systems, Tokyo, Japan), a 3D video processor (3DV-190; Olympus Medical Systems), a light source (EVIS EXERA III Xenon Light Source, CLV-190; Olympus Medical Systems), and a 3D medical display (LMD-3251MT; Sony, Tokyo, Japan; Figure 1). The 3D flexible endoscope (tip outer diameter, 12.2 mm) has two camera lenses (right and left) and a charge-coupled device at the tip of the scope (Figure 2). Images obtained through each lens are sent to each video processor as an electrical signal, which is then synthesized as a 3D image via the 3D video processor. The 3D image is visualized using the 3D monitor and 3D glasses. Stepping a foot pedal alternates the appearance of 2D and 3D images on the monitor. Similarly, with a foot pedal, white light and narrow band images can be switched. The 3D scope has a device channel which is 2.8 mm in diameter.

### 2.2. Endoscopic Submucosal Dissection

**Figure 3:**
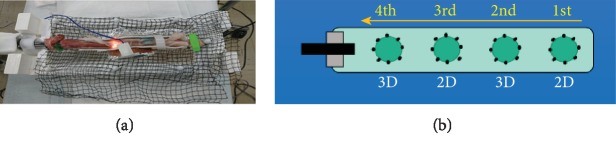
The experimental setting for the endoscopic submucosal dissection procedure. (a) The isolated porcine esophagus set-up is shown. (b) Four virtual target lesions with 15 mm diameter marked areas are lined up at equal intervals on the posterior wall of the isolated porcine esophagus.

For this study, we used isolated porcine esophagus fixed on an instrument developed for *ex vivo* ESD training (Figure 3(a)). A virtual ESD target lesion of 15 mm diameter was made by circumferential markings just outside a 15 mm diameter plastic disc with a DualKnife J (Olympus Medical Systems, Tokyo, Japan). In each isolated esophagus, four target lesions were lined up on the posterior wall (Figure 3(b)). At first, hyaluronic acid solution (Boston Scientific Japan K.K., Tokyo, Japan), colored blue with indigo carmine for contrast, was injected with a needle (25 G, 3 mm; TOP Kogyo Company, Ltd., Niigata, Japan) into the submucosa of the target lesion and surrounding area. After circumferential incision, the submucosa was dissected under direct 2D or 3D visualization using a dual knife.

Six endoscopists (3 experts and 3 trainees) participated in this study. Each endoscopist performed ESD on the 4 target lesions in one isolated esophagus as one sequential session. The ESD procedures on the 4 lesions were firstly performed under 2D vision and then under 3D and 2D visions, alternatively. All 3 experts had performed more than 300 ESD procedures, while all the trainees had performed less than 50 ESD procedures.

### 2.3. Study Endpoints

The endpoints of this study were en bloc resection rate (%), procedure time for submucosal local injection and incision/dissection (seconds), incision/dissection speed (resected area (mm^2^)/procedure time (s)), and the number of technical adverse events (perforation, muscle layer damage, or sample damage). The degree of the sense of security during ESD and the degree of fatigue and eye strain after ESD were also assessed by a visual analog scale (VAS). The VAS for sense of security had 5 grades, from 1 to 5. If the endoscopist had anxiety during the procedure, the rating was 1. If the endoscopist felt secure, the rating was 5. If there was no anxiety and also no sense of security, the rating was 3. The VAS for fatigue and eye strain also had 5 grades, from 1 to 5. A score of 1 meant that the endoscopist had no feeling of exhaustion or eye strain after the procedure, while a score of 5 meant that the endoscopist had a feeling of severe exhaustion or severe eye strain.

### 2.4. Statistical Analysis

Statistical analysis was performed with EZR (Saitama Medical Center, Jichi Medical University, Saitama, Japan), which is a graphical user interface for R version 2.13.0 (the R Foundation for Statistical Computing, Vienna, Austria). More precisely, EZR is a modified version of R commander (version 1.6-3) designed to add statistical functions frequently used in biostatistics [5]. Differences between the two groups were analyzed by *t*-tests. *P* values < .05 were considered to be statistically significant.

## 3. Results

All of the endoscopists completed the ESD procedure using both 2D and 3D visions. The en bloc resection rate was 100% for both 2D and 3D visions (Table 1).

Submucosal injection took the 3 experts, on average, 130.3 (standard deviation (SD), 20.0) seconds using 2D vison and 133.3 (29.1) seconds using 3D vision, while the 3 trainees took 177.2 (43.1) seconds with 2D vision and 181.2 (61.6) seconds with 3D vision. The incision/dissection time in experts was 510 (218.3) seconds with 2D vision and 435.5 (74.7) seconds with 3D vision and that in trainees was 955.3 (225.0) seconds for 2D vision and 927.2 (209.4) seconds for 3D vision. Therefore, the procedure times for submucosal injection and incision/dissection were equivalent between 2D and 3D vision endoscopies in both experts and trainees. The incision/dissection speed was equivalent between 2D and 3D visions; in experts, it was 0.38 (0.14) mm^2^/s for 2D and 0.39 (0.09) mm^2^/s for 3D, and in trainees, it was 0.22 (0.07) mm^2^/s with 2D and 0.22 (0.06) mm^2^/s with 3D vision (Table 1).

No perforation was observed during any ESD session. The mean number of technical adverse events (muscle layer damage or sample damage) using 2D vision was 3.5 (SD, 4.09) and using 3D vision was 1.33 (2.80) in trainees (*P* = .06) (Table 1). In experts, the number of technical adverse events using 2D vision was 0 and using 3D vision was 0.17 (0.41), meaning there was no significant difference between 2D and 3D visions.

The degree of sense of security during ESD procedures using 3D vision was 3.67 (0.82) and that using 2D vision was 2.67 (0.52) in trainees (*P* = .04) (Table 1). In experts, however, there was no significant difference in the sense of security between 2D vision (3.00 [0.00]) and 3D vision (3.83 [1.47]).

Although there was no significant difference in the degree of eye strain between 2D vision (3.00 [0.89]) and 3D vision (2.67 [1.03]) in experts, in trainees, the degree of eye strain using 3D vision (3.00 [0.00]) was significantly higher than that using 2D vision (2.17 [0.41]; *P* = .004) (Table 1). On the other hand, there was no significant difference in the degree of fatigue shown between 2D and 3D visions in both trainees and experts.

## 4. Discussion

In complicated endoscopic therapy with a high degree of difficulty, a long period of training is necessary for the acquisition of appropriate therapeutic techniques. The technical difficulty of ESD and the high rate of complications have delayed the worldwide spread of this endoscopic treatment, even though ESD achieves a high curability, compared to endoscopic mucosal resection [6]. Therefore, the challenge is to reduce the technical difficulty of ESD so endoscopists can perform the procedure even with limited experience. In the present study using an *ex vivo* model of esophageal ESD, 3D flexible endoscopy reduced technical errors (muscle layer damage and sample damage) during ESD performed by trainees, compared to 2D flexible endoscopy. In addition, 3D flexible endoscopy improved the feeling of security during ESD in a total of endoscopists. These results suggest that 3D flexible endoscopy may make ESD easier and more secure with a lower rate of adverse events, especially in trainees with limited experience.

We have already reported the feasibility of 3D endoscopy in *ex vivo* gastric ESD [4], and the present study is the first to evaluate the efficacy of 3D flexible endoscopy in esophageal ESD, compared to 2D flexible endoscopy. In the fields of surgery and gynecology, 3D laparoscopy is already used in clinical practice. There have been many studies and systematic reviews of 2D and 3D laparoscopies. Two such reviews reported that overall, 3D laparoscopy appears to improve procedure speed and reduce the number of performance errors when compared to 2D laparoscopy [1, 2]. Indeed, 63%-77% of previous studies have reported a lower rate of errors when the task is performed with 3D vision, compared with 2D vision. One research group attempted to explore the causes and prevention of laparoscopic injuries and found that the most common reason for surgical laparoscopic injuries is visual misperception [7]. The enhancement of visual depth perception provided by 3D vision may improve the quality of laparoscopic surgery and patient safety [8]. Similar to 3D laparoscopy, 3D flexible endoscopy offers visual depth perception and reduces ESD adverse events, especially in trainees, suggesting that 3D flexible endoscopy may be one of the innovations that enables endoscopists with only limited experience to perform ESD, and may even enhance the world-wide spread of ESD.

In our study, the procedure times for submucosal injection and cutting and dissection during ESD were equivalent between 2D and 3D endoscopies. In a systematic review comparing 2D and 3D laparoscopies, 71% of the 31 included trials reported significantly reduced performance times using 3D vision, compared with 2D vision [1]. The beneficial effects of 3D vision may differ among procedures. Three-dimensional vision may reduce procedure times for techniques in which precise visual depth perception is essential. Suturing is one of these procedures, and indeed, suturing performance is significantly superior under 3D laparoscopy, compared to 2D laparoscopy [9]. *Ex vivo* esophageal ESD is artificial, and there is no unexpected movement of the target lesion due to breathing or heart beats, which makes ESD more difficult in the clinical setting. Therefore, in this artificial setting, having precise visual depth perception would be unlikely to improve procedure times very much. Certainly, from the data on 3D laparoscopy, 3D flexible endoscopy has the possibility of shortening ESD procedure times in the clinical setting.

One of the drawbacks of 3D vision using a 3D stereoscopic display and 3D eye glasses is visually induced symptoms, such as eye strain, double vision, headache, dizziness, nausea, and palpitations. The visual stimulus provided by a 3D stereoscopic display differs from that of the real world because the image provided to each eye is produced on a flat surface and the distance from the screen to the eye remains fixed. As a result, unlike in the real world, the stimulus to accommodation and the stimulus to convergence do not match. This mismatch is supposed as a major cause of visually induced symptoms; however, susceptibility to these symptoms appears different among different individuals and settings. Nevertheless, 3D vision using a 3D stereoscopic display and 3D eye glasses can cause visually induced symptoms. The present study and our previous study show that 3D flexible endoscopy significantly induces eye strain in trainees, but not in experts. Almost half of the previous studies using 3D laparoscopy reported side effects, such as discomfort, dizziness, eye strain, nausea, and tiredness. These adverse side effects are one of the limitations of 3D vision using 3D stereoscopic displays and 3D eye glasses. However, 3D vision can be achieved by autostereoscopic displays, in which 3D glasses are not necessary [10]. This is one way to overcome these side effects.

One of the limitations of this study is that ESD was performed in an artificial *ex vivo* model, and the results obtained here cannot be directly applied to ESD in the clinical setting. In particular, hemorrhage may disturb 3D vision if one of the two lenses is visibly distorted due to blood adhesion, because the 3D images are constructed by processing images from the right and left lenses. In this situation, the visual disturbance can be avoided by stepping on the foot pedal and switching from 3D to 2D visions. Another limitation is that the sample size was relatively small.

In this study, we demonstrated that the newly developed 3D flexible endoscopy system may improve the sense of security during ESD and be used at least as safely as conventional 2D endoscopy system although there were some limitations. Since any disadvantages of 3D flexible endoscopy were not shown in the *ex vivo* pilot, the next step is the application of 3D flexible endoscopy in a clinical ESD setting. We are now planning to perform a clinical study of ESD under 3D vision, and this may clarify the importance of 3D endoscopy in the clinical setting.

## 5. Conclusions

3D vision improves the sense of security during ESD and may reduce technical errors, especially in trainees, indicating the feasibility of a clinical trial of ESD under 3D vision.

## Figures and Tables

**Table 1:** Outcomes using 2D and 3D visions.

	2D	3D	*P* values
All endoscopists			
En bloc resection rate (%) (*n*)	100% (12/12)	100% (12/12)	
Submucosal local injection time (s)	153.8 (40.3)	157.3 (52.3)	.819
Incision/dissection time (s)	732.7 (314.3)	681.3 (297.3)	.460
Incision/dissection speed (mm^2^/s)	0.30 (0.14)	0.30 (0.12)	.831
Adverse events (*n*)			
Perforation	0.00 (0.00)	0.00 (0.00)	
Muscle layer damage	1.42 (3.00)	0.75 (2.01)	.180
Sample damage	0.33 (0.65)	0.00 (0.00)	.104
Technical adverse events (muscle layer damage, sample damage)	1.75 (3.31)	0.75 (2.01)	.104
VAS			
Sense of security	2.83 (0.39)	3.75 (1.14)	.020^∗^
Fatigue	2.67 (0.78)	2.92 (0.79)	.463
Eye strain	2.58 (0.79)	2.83 (0.72)	.515
Trainees			
Submucosal local injection time (s)	177.2 (43.1)	181.2 (61.6)	.887
Incision/dissection time (s)	955.3 (225.0)	927.2 (209.4)	.823
Incision/dissection speed (mm^2^/s)	0.22 (0.07)	0.22 (0.06)	.965
Adverse events (*n*)			
Perforation	0.00 (0.00)	0.00 (0.00)	
Muscle layer damage	2.83 (3.82)	1.33 (2.80)	.122
Sample damage	0.67 (0.82)	0.00 (0.00)	.102
Technical adverse events (muscle layer damage, sample damage)	3.50 (4.09)	1.33 (2.80)	.063
VAS			
Sense of security	2.67 (0.52)	3.67 (0.82)	.041^∗^
Fatigue	2.33 (0.82)	2.67 (0.82)	.611
Eye strain	2.17 (0.41)	3.00 (0.00)	.004^∗^
Experts			
Submucosal local injection time (s)	130.3 (20.0)	133.3 (29.1)	.859
Incision/dissection time (s)	510.0 (218.3)	435.5 (74.7)	.386
Incision/dissection speed (mm^2^/s)	0.38 (0.14)	0.39 (0.09)	.804
Adverse events (*n*)			
Perforation	0.00 (0.00)	0.00 (0.00)	
Muscle layer damage	0.00 (0.00)	0.17 (0.41)	.363
Sample damage	0.00 (0.00)	0.00 (0.00)	
Technical adverse events (muscle layer damage, sample damage)	0.00 (0.00)	0.17 (0.41)	.363
VAS			
Sense of security	3.00 (0.00)	3.83 (1.47)	.224
Fatigue	3.00 (0.63)	3.17 (0.75)	.611
Eye strain	3.00 (0.89)	2.67 (1.03)	.638

Values are mean (SD), unless otherwise indicated. 2D: 2-dimensional; 3D: 3-dimensional; SD: standard deviation; VAS: visual analog scale. ^∗^Significant difference between 2D and 3D endoscopies.

## Data Availability

The data used to support the findings of this study are included within the article.
